# Developing a theranostic nanobody targeting FAP for cancer imaging and therapy

**DOI:** 10.1186/s41181-025-00405-z

**Published:** 2025-12-29

**Authors:** Lital Ben-Naim, Suma Prabhu, Miguel Ferreira, Shvan J. Raheem, Shadi A. Esfahani, Umar Mahmood, Pedram Heidari

**Affiliations:** 1 Division of Nuclear Medicine and Molecular Imaging, Department of Radiology, Massachusetts General Hospital, Harvard Medical School, 55 Fruit St, White 427J, Boston, MA 02114 USA; 2 Center for Precision Imaging, Division of Nuclear Medicine and Molecular Imaging, Department of Radiology, Massachusetts General Hospital, 55 Fruit St, White 427J, Boston, MA 02129 USA

**Keywords:** Fibroblast activation protein (FAP), Nanobody (Nb), Radiotheranostics, PET imaging, Targeted radiotherapy

## Abstract

**Background:**

Fibroblast activation protein (FAP) is a pan-cancer target. Its selective expression on the majority of solid tumors with minimal to absent expression in healthy tissues positions FAP as a promising target for radiotheranostic applications. Nanobodies (Nbs) have unique characteristics, including small size, high affinity, stability, and ease of modification, making them ideal candidates for cancer diagnostics and targeted radiotherapeutics. Llama-derived Nbs were generated and screened against full-length FAP, with three unique candidates selected from the library for further characterization. The lead candidate Nb159 was engineered for site-specific radiolabeling with ^89^Zr for PET imaging and with ^177^Lu coupled with PEG for therapeutic evaluation in mice bearing FAP-positive U87 tumor xenografts.

**Results:**

Nb159 exhibited exceptional picomolar binding affinity to FAP with stable interaction and slow dissociation. PET imaging with [^89^Zr]Zr-Nb159 demonstrated specific tumor uptake, peaking at 1 h post-injection, with rapid renal clearance and minimal uptake in non-target organs. A competitive binding study confirmed its specificity to FAP on U87 tumors, as pre-injection with a tenfold molar excess of unlabeled Nb159 reduced tumor uptake by ~ 55% (3.78 ± 0.50 to 1.67 ± 0.26%ID/g). PEGylation of Nb159 improved its pharmacokinetic profile, yielding prolonged tumor accumulation and significantly reduced renal retention when co-injected with lysine. PET imaging further demonstrated target-specific uptake in FAP-positive U87 xenografts, which exhibited higher signal than FAP-negative HCT116 tumors, with SUV_mean_ at 48 h of 0.45 ± 0.04 versus 0.09 ± 0.01 (*P* < 0.0001). In the therapeutic study, [^177^Lu]Lu-PEG-Nb159 demonstrated significant tumor growth inhibition with no observable toxicity. Mice treated with a single dose of [^177^Lu]Lu-PEG-Nb159 survived significantly longer compared to either [^177^Lu]Lu-DOTA (23 days, *P* < 0.001, HR: 0.06107) or vehicle (21 days, *P* < 0.0001, HR: 0.04017).

**Conclusions:**

The lead candidate Nb159 holds promise as a versatile platform for FAP-targeted radiotheranostics, with [^89^Zr]Zr-Nb159 serving as an effective companion diagnostic and [^177^Lu]Lu-PEG-Nb159 demonstrating promising therapeutic potential. These findings support further development of Nb159-based radiopharmaceuticals for treatment of FAP positive tumors.

**Supplementary Information:**

The online version contains supplementary material available at 10.1186/s41181-025-00405-z.

## Introduction

Radiotheranostics has become a cornerstone of precision oncology in recent years (Bodei et al. 2022). By coupling diagnostic imaging agents alongside therapeutic counterparts that release particulate radiation, radiotheranostics enables a tightly coupled, patient-specific approach to cancer care (Zhai and Bobba 2024). Fibroblast activation protein (FAP) is a transmembrane serine protease with high expression on cancer-associated fibroblasts (CAFs) in more than 90% of epithelial carcinomas (Altmann et al. 2021; Dziadek et al. 2024) and on some tumor cells of mesenchymal origin (Crane et al. 2023). Its highly selective expression, with minimal to no presence in healthy tissues, makes FAP an attractive target for radiotheranostic applications (Lindner et al. 2019; Thapa et al. 2024).

In recent years, a variety of FAP-targeted PET tracers have been developed, mostly quinoline-based FAP inhibitors (FAPIs). Certain FAPI analogs have demonstrated improved tumor visualization and diagnostic accuracy in specific clinical applications compared to ^18^F-FDG (Pang et al. 2022). Despite their applications in clinical settings, most small molecule FAPI analogs exhibit limited tumor uptake, rapid clearance, and partial hepatobiliary excretion, which can diminish and limit the therapeutic window. Specifically, poor tumor retention remains a challenge for many FAP-targeted radiopharmaceuticals, hindering optimal therapeutic outcomes (Lindeman et al. 2022). To address this, various strategies such as introducing albumin-binding moieties or generating multivalent tracers have enhanced tumor retention but sometimes at the cost of increased nonspecific uptake and potential radiation-induced toxicity. Thus, improving tumor affinity and retention while maintaining favorable pharmacokinetic profiles and minimizing off-target deposition is central to developing more effective FAP-targeted radiotheranostics. Nanobodies (single-domain antibodies, VHHs) occupy an optimal space between small molecules and antibodies and provide an attractive alternative to address the tracer retention challenge. Nbs (~ 15 kDa), derived from Camelid immunization, possess exceptional target affinity, stability, and tissue penetration. Their modular structure allows precise customization for site-specific labeling and optimization of pharmacokinetics (Alexander and Leong 2024). Furthermore, their excretion, primarily through the urinary tract, is favorable for therapy.

In this study, we describe the generation of an immune library, screening against transmembrane FAP, and characterizing the lead candidate—Nb159. We present its diagnostic potential in PET imaging, pharmacokinetic optimizations for targeted radiotherapy, and therapeutic efficacy.

## Materials and methods

### Transduction of transmembrane FAP in Camelid-origin Cells

Stable Dubca cells (Klopries et al. 1995) overexpressing cell surface human FAP (hFAP) were prepared for Llama immunization and screenings, as detailed in the ‘supplemental materials.’ Further methods on Nb library construction, screening, and selection of candidates, as well as in vivo imaging studies are described in the supplementary file for comprehensive experimental details.

### Llama immunization with FAP-Dubca cells

To induce a humoral immune response to cell-surface human FAP, a female Llama was immunized subcutaneously weekly six times with approximately 1 × 10^8^ FAP-Dubca cells suspended in 1.5 ml of sterile PBS. To avoid receptor denaturation, in the first three immunizations, cells and Incomplete Freund’s adjuvant were injected 5 cm apart. For the subsequent immunizations, cells were gently mixed with Gerbu P adjuvant (Gerbu Biotechnics) at equal volumes to 3 ml. The immune response against FAP was monitored following the third injection, as previously described (Chow et al. 2019). Four days after the final immunization, 100 ml of peripheral blood was collected from the jugular vein. All animal experiments were conducted under institutional guidelines and approved by the Institutional Animal Care and Use Committee (IACUC).

### Recombinant expression and purification

Three anti-FAP Nbs candidates, Nb17, Nb132, and Nb159, were engineered to include a C-terminal Sortase recognition motif and 6 × His tag (LPETGGHHHHHH). Nbs were expressed in WK6 *E. coli* cells (ATCC) as previously described (Pardon et al. 2014). Soluble His-tagged Nbs were extracted from the periplasm and purified by Ni–NTA beads (Invitrogen) in a gravity column (Pishesha et al. 2021), and were eluted with 15 ml of 500 mM Imidazole in PBS. Yields ranged from 5–15 mg/ml as determined by a nano-spectrophotometer (Denovix DS-11). The purity of recombinant Nbs was confirmed by SDS-PAGE gel electrophoresis, and molecular mass was analyzed by mass-spectrometry MALDI-TOF.

### Flow cytometry

Cell-surface FAP expression was evaluated in U87, CAFs, and HCT116, using PE-conjugated anti-FAP antibody (R&D Systems). To evaluate the binding of Nbs to cell-surface FAP, cells were incubated with Nb17, Nb132, or Nb159 (500 nM), followed by incubation with iFluor 647 anti-Camelid VHH antibody (GenScript). Data were acquired using the Flow Cytometer BD LSRFortessa X-20 (BD Biosciences) and analyzed with FlowJo v10.8 Software.

### Binding affinity measurements

The binding affinity of Nbs to hFAP was determined by surface plasmon resonance (SPR) using a Biacore T200 (Cytiva). Recombinant FAP protein (Biolegend) diluted in 10 mM sodium acetate pH 5 was immobilized on a CM5 chip (Cytiva) according to the manufacturer’s instructions. Nbs were tested in a two-fold serial dilution (6.25–0.0976 nM) at a flow rate of 30 μl/min. Sensorgrams were corrected with appropriate blank references and fitted with a 1:1 binding model to calculate affinity constants using Biacore Evaluation Software V2.0.

### Site-specific functionalization of Nbs

The site-specific conjugation of Nbs was carried out via Sortase (SrtA) enzyme-mediated reaction as previously described (Teunissen et al. 2021). The reaction mixture, composed of 7 µM SrtA-7M, 2–4 mg/ml Nb-LPETGG-HHHHHH, and 1–2 mM of nucleophilic substrate, proceeded overnight at 4 °C, followed by purification using PD-10 column (Cytiva) and Ni–NTA beads in a microcentrifuge tube. Three nucleophilic substrates with a chelating group were used for C-terminal conjugation in this study: Gly_3_-Deferoxamine (DFO), Gly_3_-PEG_3_-Lys(Azide)-PEG_3_-DFO (GenScript), and Gly_3_-PEG_3_-Lys(Azide)-PEG_3_-K(DOTA) (Anaspec). Nbs conjugated with Azide-chelator substrates (1–2 mg/ml) were further coupled to polyethylene glycol (PEG) with threefold molar excess of DBCO-PEG 20 kDa (PEG; Vector Laboratories) through a Cu-free click reaction for 2 h at 4 °C.

### Radiolabeling

For PET imaging and biodistribution studies, DFO-Nb159 and PEG-DFO-Nb159 were labeled with Zirconium-89 (^89^Zr), as follows: 111 MBq of ^89^Zr-oxalate solution (3D Imaging) was diluted with 300 µl of 1 M HEPES and added to 200 µl of DFO-functionalized Nb (1–2 mg) in HEPES/NaCl buffer (pH 7.5), adjusted to pH 6.8–7.5, and incubated for 1 h at room temperature. Radiochemical purity was assessed by instant thin-layer chromatography (iTLC) with 50 mM DTPA (MilliporeSigma) as the mobile phase. For radiotherapy study, PEG-DOTA-Nb159 or Gly_3_-PEG_3_-Lys(Azide)-PEG_3_-K(DOTA) substrate was labeled with Lutetium-177 (^177^Lu) as follows: 370 MBq of ^177^Lu-chloride solution (Oak Ridge National Laboratory) was diluted with 200 µl of 1 M ammonium Acetate pH 5, supplemented with 3.5 mM ascorbic acid and 3.5 mM gentisic acid (MilliporeSigma), and added to 200 µl of 2 mg DOTA-functionalized Nb or substrate, in HEPES/NaCl buffer. The reaction pH was adjusted to 5–5.5, and incubated for 1 h at 37 °C. The radiolabeled products were purified using PD-10 columns pre-equilibrated with PBS, and radiochemical purity was assessed by iTLC with 50 mM EDTA (MilliporeSigma) as the mobile phase.

### Optimization of Nb pharmacokinetics

To optimize tumor retention and minimize renal accumulation of radiolabeled Nbs, we compared the biodistribution of [^89^Zr]Zr-Nb159 (~ 15 kDa) and its PEGylated counterpart [^89^Zr]Zr-PEG-Nb159 (~ 36 kDa) in the presence or absence of L-lysine (EMD Millipore). Female athymic nude mice (Charles River Laboratories) 4–6 weeks-old (n = 12, weight: 25 ± 3.7 gr), were subcutaneously inoculated in the upper right flank with 1 × 10^6^ FAP^+^ U87 cells suspended in PBS/Matrigel (1:1, 100 μl). Mice were randomly assigned to four groups (n = 3/group) and i.v. injected with radiolabeled Nbs (approximately 6.29 MBq, 100 μl) into the lateral tail vein. Groups 1 & 2 were injected with [^89^Zr]Zr-Nb159 diluted in saline or lysine solution (20 mg from a 400 mg/ml stock). Groups 3 & 4 were injected with [^89^Zr]Zr-PEG-Nb159 diluted in saline or lysine solution. PET imaging was conducted on a 4.7-Tesla MR/PET scanner (Bruker) at 1, 6, and 24 h p.i. Mice were anesthetized with 2% isoflurane, and static PET images were acquired for 20 min, with MR images captured for anatomical reference. Images were reconstructed using a 3D-MLEM algorithm and processed using PMOD software. VOIs were manually drawn over specific tissues (tumor, heart, kidneys) to calculate PET standard uptake value (SUV).

### In vivo pharmacokinetics of [.^89^Zr]Zr-PEG-Nb159

Female nude mice (weight: 24 ± 1.40 gr) bearing FAP-positive U87 or FAP-negative HCT116 tumor xenografts (200–250 mm^3^) were injected via tail vein with [^89^Zr]Zr-PEG-Nb159 (8.76 ± 0.62 MBq) mixed with 20 mg L-lysine in 100 µl total volume (n = 6/group). Mice were imaged using the Argus PET scanner (Sedecal), at 1, 6, 24 and 48 h p.i for 20 min in two bed positions. PET images were reconstructed using 3D Ordered Subsets Expectation–Maximization (OSEM) and imported into PMOD software for processing and SUV analysis.

### Radiopharmaceutical therapy with [.^177^Lu]Lu-PEG-Nb159

For radiopharmaceutical therapy (RPT), female nude mice bearing U87 tumors (125–167 mm^3^) were randomly categorized into 3 groups (n = 10/group): the treatment group was i.v. injected with a single dose of [^177^Lu]Lu-PEG-Nb159 (18.5 ± 1.07 MBq), the control group received a single dose of labeled chelator [^177^Lu]Lu-DOTA (18.79 ± 0.99 MBq), and the third group was injected with saline as a vehicle solution. All groups were co-administered with 20 mg lysine to reduce kidney uptake. Mice were monitored regularly for weight, welfare, and tumor volume. Tumor dimensions were measured 2–3 times a week by a digital caliper (Volume = Length × Width^2^ × 0.52). The endpoint criteria for the survival study included tumor volume exceeding ~ 1500 mm^3^, ulcered tumors, or > 20% weight loss.

### Serum stability assay

Radiolabeled conjugates, [^177^Lu]Lu-PEG-Nb159 (18.5 MBq) and [^89^Zr]Zr-PEG-Nb159 (3.7 MBq), were incubated in 500 µl of mouse serum or PBS at 37 °C with agitation (350 rpm) for 7 days. Daily aliquots were collected in triplicate and analyzed by iTLC to assess complex stability.

### Statistical analysis

All statistical analyses were performed using GraphPad Prism 9.4.1. Kaplan–Meier curve was analyzed using the Gehan-Breslow-Wilcoxon test. Differences among groups were compared using Student’s t-test or one-way analysis of variance (ANOVA). Results are depicted as mean ± SD; statistical significance was considered at *P*-values < 0.05. Values are indicated by asterisks in the figures as followed: *P < 0.05, **P < 0.01, ***P < 0.001, ****P < 0.0001, and n.s. = non-significant.

## Results

### In vitro binding measurements

**Fig. 1:**
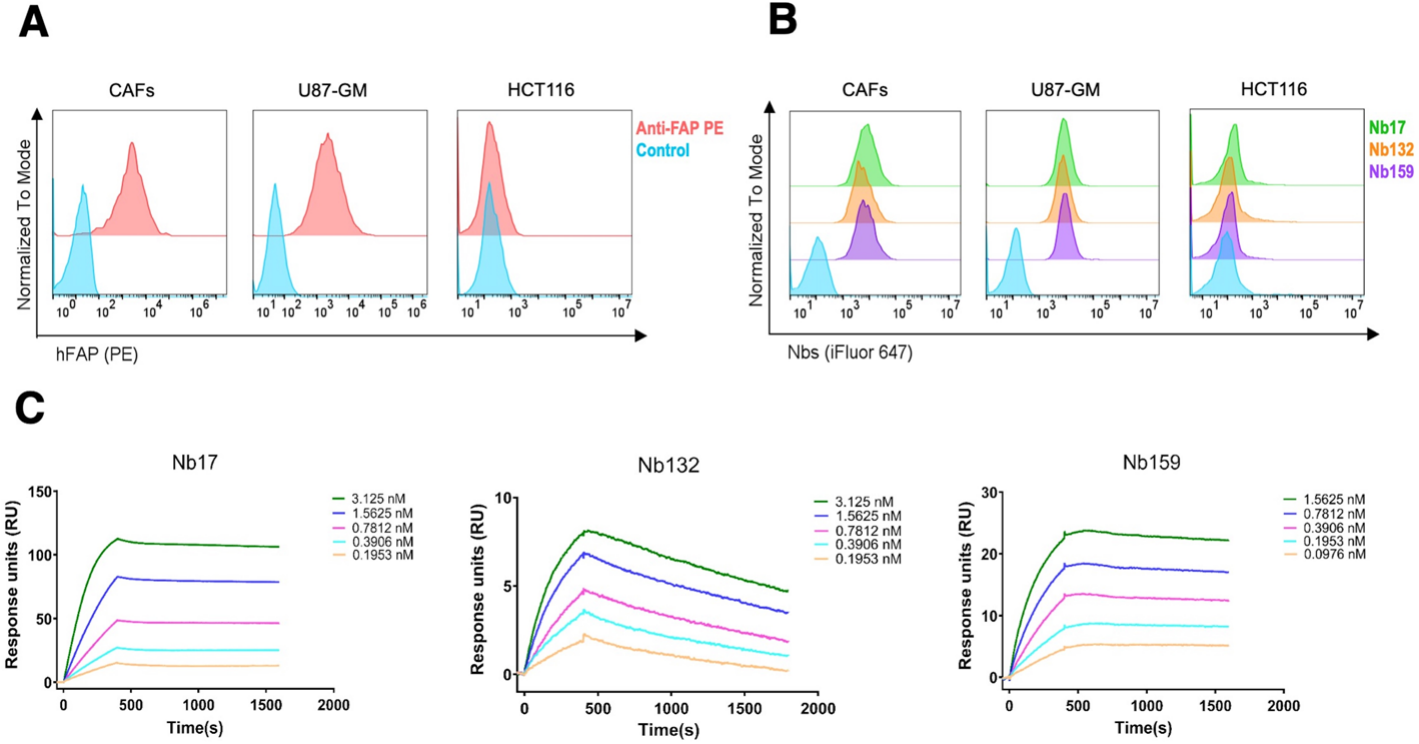
Binding measurements of Nbs to FAP. **A** Flow-cytometry analysis confirmed the expression levels of FAP on CAFs (Colorectal tumor origin), U87 glioblastoma, and HCT116 colorectal cell lines. **B** All 3 Nbs exhibited specific binding to FAP-expressing cells, CAFs and U87 with no binding observed in HCT116 cells. **C** Representative SPR sensorgrams show the response (RU) as a function of time during the association and disassociation binding phases of Nb17, Nb132, and Nb159 to immobilized recombinant hFAP. A 1:1 kinetic model was used to calculate the affinity (K_D_)

Llama-derived Nbs were selected following immunization with stable FAP-expressing Dubca cells (Fig. S1) and subsequent screening as detailed in supplemental information, with three candidates, Nb17, Nb132, and Nb159, chosen for their distinct CDR lengths and sequences. The Nbs were purified (Fig. S2) and subjected to flow-cytometry analysis to evaluate binding to native membrane-bound FAP across three cell lines: CAFs, U87 (FAP-positive), and HCT116 (FAP-negative). FAP expression was high in CAFs and U87, with no detectable expression in HCT116 cells (Fig. 1A). Accordingly, all three Nbs at 500 nM demonstrated specific binding to surface FAP on CAFs and U87, with no binding observed in HCT116 cells (Fig. 1B). Binding kinetics to recombinant hFAP were further analyzed by SPR, revealing picomolar binding affinities with variation among the Nbs (Fig. 1C, Table 1). Notably, cross-reactivity analysis demonstrated that Nb159, Nb132, and Nb17 bind specifically to human FAP with no detectable cross-reactivity to murine FAP (Fig. S3).

**Table 1:** SPR Kinetic binding constants for the interaction of 3 Nbs to FAP

Analyte	K_on_ [M^−1^ S^−1^]	K_off_ [S^−1^]	K_D_ (pM)
Nb17	3.23E + 6 ± (3.20E + 3)	4.15E-5 ± (1.10E-7)	12.8
Nb132	2.27E + 6 ± (2.23E + 3)	4.90E-4 ± (5.70E-7)	215.5
Nb159	5.25E + 6 ± (4.10E + 3)	5.33E-5 ± (5.20E-7)	10.1

K_on_ = association rate constant, K_off_ = dissociation rate constant, K_D_ = equilibrium dissociation constant

### In vivo evaluation of [^89^Zr]Zr-Nb159 Specificity for FAP

Building upon the fluorescence imaging results, Nb159 displayed higher in vivo tumor uptake and retention compared to Nb17 and Nb132 (Fig. S4B–D, S5) and was therefore selected as the lead candidate for further in vivo evaluation. Nb159 was site-specifically radiolabeled with ⁸⁹Zr (t_1/2_ = 3.3 d) achieving a radiochemical purity > 97% and a decay-corrected yield of 81.8%. To evaluate its specificity for FAP, [⁸⁹Zr]Zr-DFO-Nb159 ([⁸⁹Zr]Zr-Nb159) was tested in U87 tumor-bearing mice using a competitive blocking study (Fig. S6). PET imaging showed that pre-treatment with a tenfold molar excess of unlabeled Nb159 significantly reduced tumor uptake to 1.67 ± 0.26%ID/g, compared to 3.78 ± 0.50%ID/g in the non-blocked group (*P* < 0.01). This ~ 55% decrease in specific binding, confirms the FAP-specific targeting of [⁸⁹Zr]Zr-Nb159.

### Comparing the pharmacokinetics of [^89^Zr]Z-Nb159 and [.^89^Zr]Z-PEG-Nb159

**Fig. 2:**
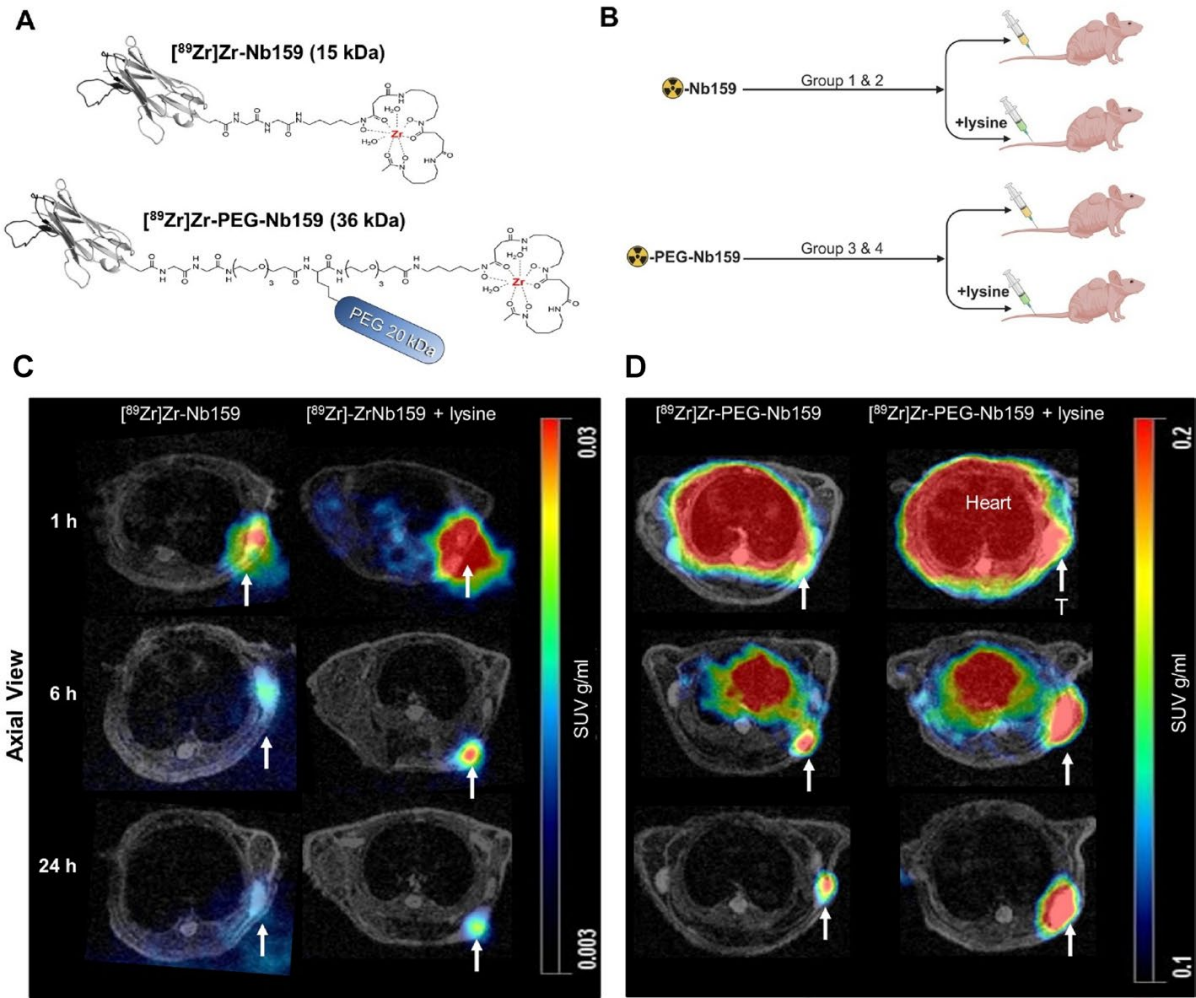
PET imaging of [^89^Zr]Zr-Nb159 and [^89^Zr]Zr-PEG-Nb159. **A** Schematic illustration of Nb159 site-specifically conjugated to DFO chelator, coupled with PEG 20 kDa, and labeled with ^89^Zr. **B** Experimental setup for comparing the pharmacokinetics of two Nb tracers in tumor-bearing mice with or without co-injection of lysine to evaluate its effect on renal uptake. **C**-**D** Representative static PET/MR images (cross section in axial view) of FAP^+^ tumor-bearing mice after i.v. injection of [^89^Zr]Zr-Nb159 or [^89^Zr]Zr-PEG-NB159, with or without lysine at 1, 6, and 24 h p.i. (n = 3/group) showing specific activity in the tumor site (marked by a white arrow, T = tumor)

**Fig. 3:**
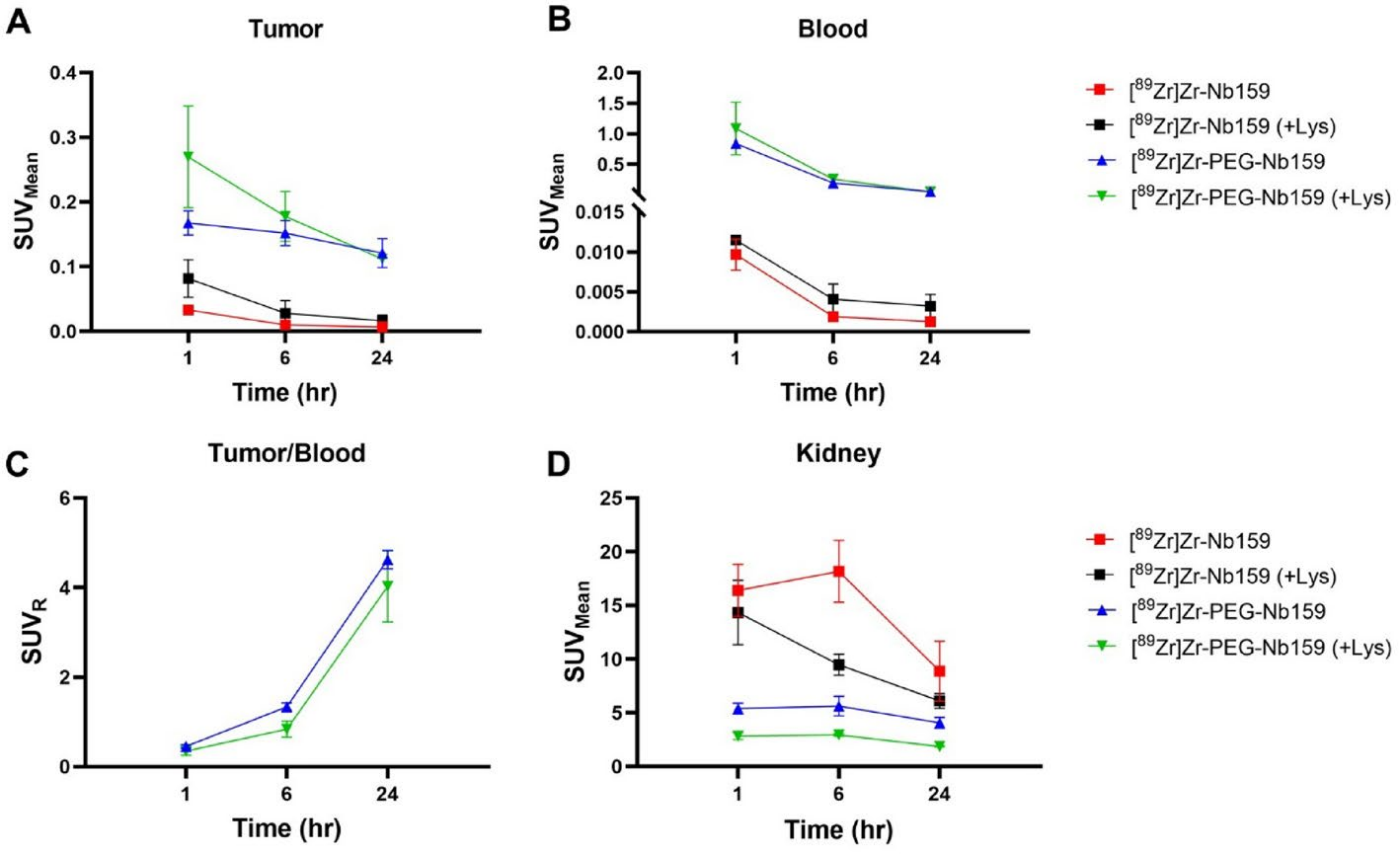
PET SUV analysis of [⁸⁹Zr]Zr-Nb159 and [⁸⁹Zr]Zr-PEG-Nb159 in U87 tumor-bearing mice. **A** Tumor SUV_mean_ over time comparing [⁸⁹Zr]Zr-Nb159 and [⁸⁹Zr]Zr-PEG-Nb159, with or without lysine co-injection. PEGylation resulted in significantly higher tumor uptake at all time points. **B** Cardiac blood pool SUV_mean_, showing prolonged circulation of PEGylated [⁸⁹Zr]Zr-Nb159 compared to the rapid clearance of unmodified [⁸⁹Zr]Zr-Nb159, with lysine co-injection exerting a modest effect on both constructs. **C** Tumor-to-blood ratio (SUV_R_ = Tumor_max_/Heart_mean_ ratio) of [⁸⁹Zr]Zr-PEG-Nb159, demonstrating increased tumor contrast over time. **D** Kidney SUV_mean_, showing markedly reduced renal uptake with PEGylated [⁸⁹Zr]Zr-Nb159 and further reduction with lysine co-injection at all time points. presented as mean ± SD

Radiolabeled Nb159 exhibited expected in vivo features of short blood half-life and fast renal clearance (Bao et al. 2021). To increase the plasma residence time and lower the renal uptake, Nb159 was modified by coupling to a 20 kDa PEG moiety (PEG-Nb159). Comparative PET analysis of [^89^Zr]Zr-Nb159 (~ 15 kDa) and [^89^Zr]Zr-PEG-Nb159 (~ 36 kDa) was conducted in U87 tumor xenografts, incorporating lysine co-injection to evaluate its impact on kidney retention. PET imaging at 1, 6, and 24 h p.i, revealed distinct uptake profiles between the two Nb constructs (Fig. 2C, D). [^89^Zr]Zr-Nb159 exhibited its highest tumor uptake at 1 h with a gradual decrease over 24 h, while co-injection with lysine resulted in a modest and non-significant increase in tumor uptake (Fig. 3A). The blood pool signal was minimal throughout the imaging time points, consistent with the rapid clearance profile of non-modified Nb159 (Fig. 3B). In contrast, [^89^Zr]Zr-PEG-Nb159 demonstrated a significantly higher tumor SUV_mean_ compared to [^89^Zr]Zr-Nb159 at all imaging time points (1 h: 0.167 ± 0.033 vs. 0.033 ± 0.003, *P* < 0.01, at 6 h: 0.152 ± 0.034 vs. 0.010 ± 0.003, *P* < 0.01, and at 24 h: 0.121 ± 0.039 vs. 0.007 ± 0.002, *P* < 0.01, without lysine) (Fig. 2D, 3A). PEGylated Nb159 demonstrated prolonged blood pool circulation with increasing tumor-to-blood ratios SUV_R_, ultimately achieving high contrast at 24 h p.i. (without lysine at 1 h: 0.452 ± 0.069, at 6 h: 1.334 ± 0.151, and at 24 h: 4.626 ± 0.358, with lysine at 1 h: 0.3469 ± 0.161, at 6 h: 0.837 ± 0.3145, and at 24 h: 4.026 ± 1.376) (Fig. 3C). Moreover, when co-injected with lysine, PEGylated Nb159 significantly reduced renal retention compared to [^89^Zr]Zr-Nb159, with reductions of 80.2% at 1 h (2.83 ± 0.57 vs. 14.3 ± 5.18 SUV_mean_, *P* < 0.01), 69.1% at 6 h (2.93 ± 0.23 vs. 9.47 ± 1.71 SUV_mean_, *P* < 0.01), and 69.5% at 24 h (1.86 ± 0.05 vs. 6.11 ± 1.21 SUV_mean_, *P* < 0.01) (Fig. 3D). These results highlight the advantage of PEGylation in improving tumor retention and reducing off-target kidney uptake.

### Tumor and off-target organ retention of [⁸⁹Zr]Zr-PEG-Nb159

**Fig. 4:**
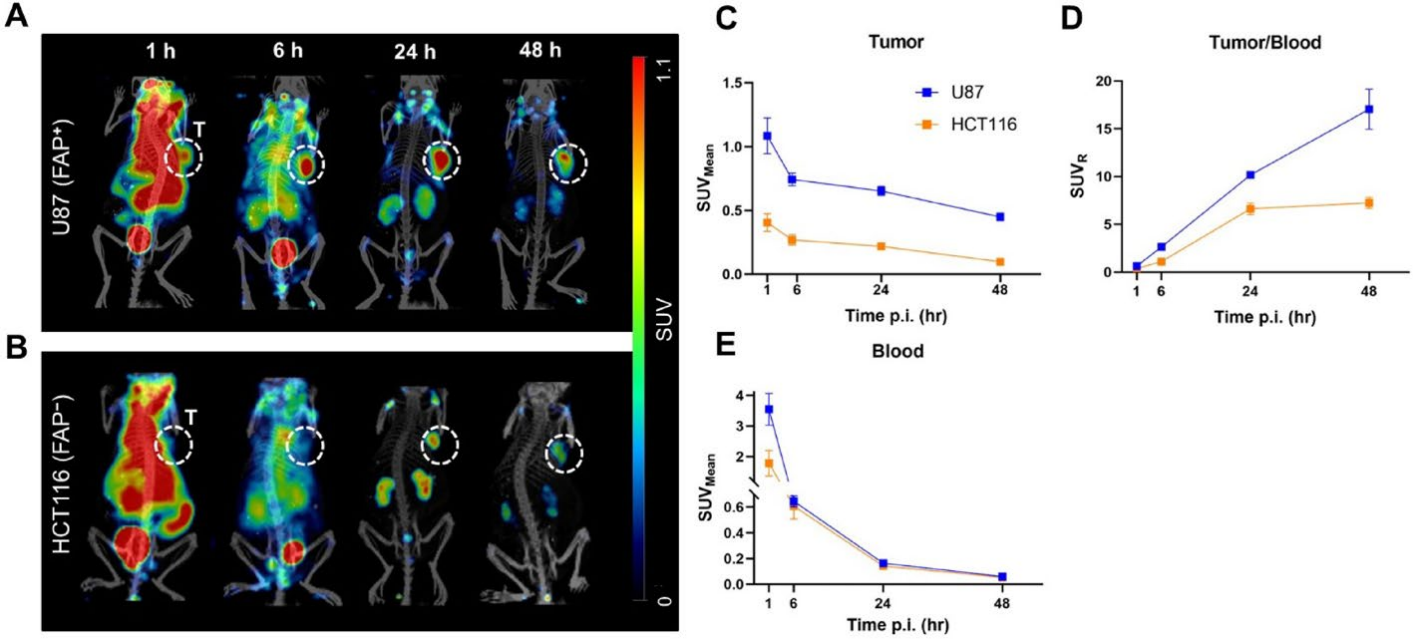
PET imaging of [^89^Zr]Zr-PEG-Nb159. Representative PET/CT images of nude mice bearing **A** U87 or **B** HCT116 xenografts at 1, 6, 24, and 48 h after co-injection of [⁸⁹Zr]Zr-PEG-Nb159 with lysine solution. Tumor site is marked by white dotted circle. **C** Tumor SUV_mean_ and **D** tumor-to-blood ratio SUV_R_ demonstrate sustained uptake and retention in U87 tumors compared to a significantly lower uptake in HCT116. **E** Blood clearance profiles show gradual elimination from circulation in both groups over time. Data represent mean ± SD (n = 6/group)

To further evaluate the in vivo performance of PEGylated Nb159, female nude mice bearing FAP-positive U87 or FAP-negative HCT116 xenografts (200–250 mm^3^, n = 6/group) were administered 8.76 ± 0.62 MBq of [^89^Zr]Zr-PEG-Nb159 supplemented with 20 mg L-lysine. Prior in vitro stability assessments confirmed that [^89^Zr]Zr-PEG-Nb159 maintained high stability throughout a 7-day incubation period, with > 94% integrity in PBS and 89% in mouse serum (Fig. S7). Mice were imaged by PET/CT at 1, 6, 24, and 48 h p.i. (Fig. 4). In U87-bearing mice, focal tumor uptake was clearly visualized from 1 h and persisted through 48 h. HCT116 xenografts exhibited markedly lower tumor uptake compared to U87 at all time points. After blood pool clearance, only low-level signal was detectable in HCT116 tumors, which declined further at later imaging points and is most likely attributable to the enhanced permeability and retention (EPR) effect rather than specific tracer binding (Heneweer et al. 2011) (Fig. 4A and B). Quantitative analysis confirmed significantly higher tumor SUV_mean_ values in U87 xenografts compared to HCT116 at all time points (Fig. 4C). At 1 h p.i. SUV_mean_ reached 1.08 ± 0.34 in U87 versus 0.37 ± 0.09 in HCT116 (*P* < 0.01). At 6 h, values were 0.74 ± 0.12 vs. 0.26 ± 0.09 (*P* < 0.01). By 24 h, the proportional difference became more pronounced, with U87 tumors exhibiting three-fold higher SUV_mean_ (0.65 ± 0.08 vs. 0.21 ± 0.03, *P* < 0.0001), which further widened at 48 h (0.45 ± 0.04 vs. 0.09 ± 0.01, *P* < 0.0001). Target selectivity is further supported by steadily increasing tumor-to-blood ratios (Fig. 4D). As background activity clears, the difference between groups became more pronounced at later time points. At 24 h, U87 xenografts reached an SUV_R_ of 10.17 ± 0.50 compared to 6.62 ± 1.34 in HCT116 (*P* < 0.001), and by 48 h, U87 further increased to 17.04 ± 4.69 versus 7.24 ± 1.14 in HCT116 (*P* < 0.01). These findings are consistent with the pharmacokinetic profile shown in Fig. 3C, where PEGylated Nb159 demonstrated prolonged blood circulation and progressive increases in tumor-to-blood contrast over time. Blood activity declined over time in both groups (Fig. 4E). At 1 h, SUV_mean_ values were slightly higher in U87-bearing mice (3.55 ± 1.27) compared to HCT116 (1.78 ± 1.02, *P* < 0.05), but no significant differences were observed thereafter (Fig. 4E). SUV_mean_ values decreased markedly by 24 h, indicating efficient clearance of the ~ 36 kDa PEGylated Nb from circulation. Ex vivo biodistribution of [⁸⁹Zr]Zr-PEG-Nb159 (Fig. S8 A and B) from an independent cohort (n = 3/group) confirmed the PET findings. Organ activity was measured at 24 and 48 h p.i. (%ID/g) across tumors, kidneys, and selected organs. The tracer showed predominant renal clearance and low retention in most other tissues. U87 tumors displayed higher and sustained uptake compared to HCT116, consistent with the in vivo PET results.

### FAP-targeted radiotherapy with [.^177^Lu]Lu-PEG-Nb159

**Fig. 5:**
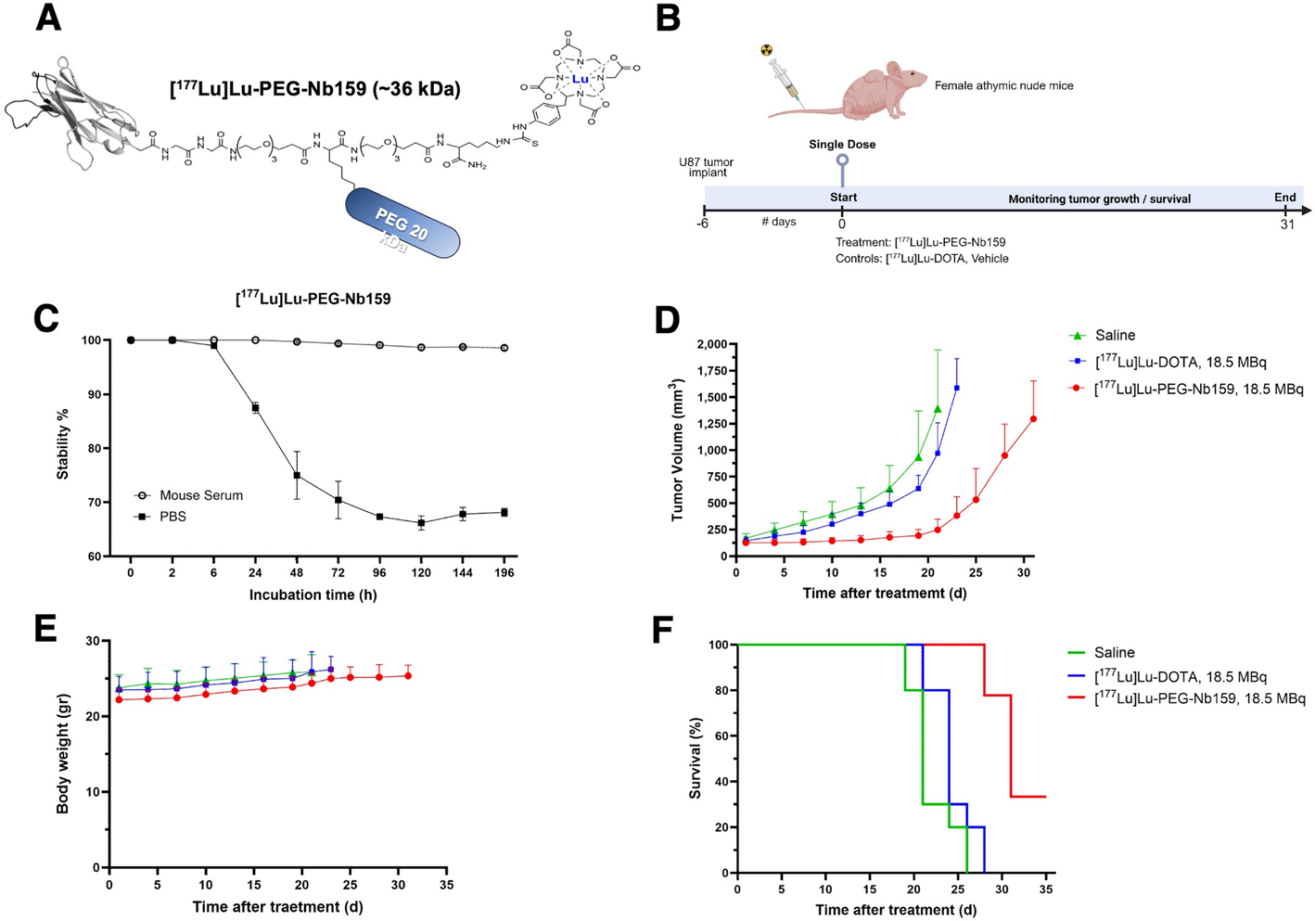
Therapeutic efficacy of [^177^Lu]Lu-PEG-Nb159. **A** Schematic illustration of Nb159 site-specifically conjugated to DOTA chelator and labeled with ^177^Lu. **B** Evaluation of therapeutic potential of [^177^Lu]Lu-PEG-Nb159 (18.5 MBq), [^177^Lu]Lu-DOTA (18.5 MBq), or saline evaluated in U87 tumor–bearing mice (n = 10/group). **C** In vitro stability of [^177^Lu]Lu-PEG-Nb159 in mouse serum or PBS over 7 days (Mean ± SD). **D** Tumor growth monitoring in U87 tumor models for treatment and control groups. **E** Changes in body weight in each group for the duration of the study. **F** Kaplan–Meier survival curvesindicate a significant improvement in survival for the [^177^Lu]Lu-PEG-Nb159 group compared to [^177^Lu]Lu-DOTA control (*** *P* < 0.001) or to saline (**** *P* < 0.0001)

To evaluate the therapeutic efficacy of Nb159, PEG₂₀-DOTA-Nb159 (PEG-Nb159) was site-specifically radiolabeled with ^177^Lu (t_1/2_ = 6.65 d), achieving a radiochemical purity of 92.97% and a yield of 94.6%. Stability assessments demonstrated high stability of [^177^Lu]Lu-PEG-Nb159 in mouse serum, retaining > 98% integrity throughout a 7-day incubation period. In contrast, incubation in PBS resulted in decreased stability over time (Fig. 5C). U87 tumor-bearing mice (125–167 mm^3^) were i.v. injected with a single dose of either [^177^Lu]Lu-PEG-Nb159 (18.5 ± 1.07 MBq), [^177^Lu]Lu-DOTA (18.79 ± 0.99 MBq, non-specific control), or saline (vehicle), co-injected with 20 mg of L-lysine to minimize renal retention. As shown in Fig. 5D, treatment with [^177^Lu]Lu-PEG-Nb159 resulted in a significant tumor growth delay with a single dose, especially noted within the first 19 days post-injection. Tumor volumes at day 19 were significantly smaller in the [^177^Lu]Lu-PEG-Nb159 group compared to the [^177^Lu]Lu-DOTA control (194.65 ± 56.77 vs. 638.04 ± 125.48 mm^3^, *P* < 0.0001) and the vehicle group (194.65 ± 56.77 vs 936.75 ± 433.51 mm^3^, *P* < 0.0001).

In contrast, tumors in the control groups progressed rapidly, reaching humane endpoints by day 21 (vehicle) or 23 ([^177^Lu]Lu-DOTA). No significant differences in body weight were observed across all groups, and no signs of toxicity were detected (Fig. 5E). Mice treated with [^177^Lu]Lu-PEG-Nb159 completed the 31-day study without weight loss and survived significantly longer compared to [^177^Lu]Lu-DOTA (*P* < 0.001, HR: 0.06107, 95% CI: 0.01532 to 0.2434) or vehicle group (*P* < 0.0001, HR: 0.04017, 95% CI: 0.009545 to 0.1690) (Fig. 4F).

## Discussion

In this work, we developed and characterized Llama-derived Nbs targeting human FAP, a key cancer biomarker for diagnosis and therapy. Cell-based immunization and library screenings were applied to generate and isolate conformation-based Nbs that recognize FAP transmembrane receptors in their native form. Three FAP-specific Nbs (Nb17, Nb132, and Nb159) with distinct CDR sequences were selected and purified. All three recognize the extracellular domain of hFAP and demonstrate strong binding affinity to FAP in the picomolar range. Their high affinity correlates to their retention in FAP^+^ tumors, as validated by in vivo fluorescent imaging. Furthermore, the Nbs demonstrated selective binding to human FAP without cross-reactivity to murine FAP, supporting their target specificity. This ensures that in vivo studies in xenograft models directly reflect human FAP targeting. Nb159 was selected as the lead candidate for subsequent in-vivo studies based on its overall optimal properties, including 10.1 pM binding affinity to FAP, the highest-affinity biomolecule reported to date in the literature.

Several classes of FAP-targeting agents, including antibodies (Hintz et al. 2020), small molecules (Lindner et al. 2018; Millul et al. 2021), and peptides (Zboralski et al. 2022), have been developed and evaluated as radiopharmaceuticals in preclinical and clinical studies (Privé et al. 2023). To date, the most encouraging clinical data is attributed to FAP-inhibitors (FAPI); FAPI-04 and FAPI-46, demonstrating imaging of solid tumors and metastases in patients (Kratochwil et al. 2019; Chandekar et al. 2023). However, the short tumor retention time of FAPI remains the limiting factor for therapy application, highlighting the need for new tracers better suited for theranostic purposes.

Nbs (12–15 kDa) occupy an optimal space between small molecules and antibodies and thus represent an attractive alternative approach. Their unique features, including small size, high solubility, stability, binding affinity, and excellent tissue penetration, make them well-suited for therapeutic applications (Jumapili et al. 2023). Furthermore, the modular nature of Nbs enables precise customization of their properties for therapeutic applications (Kumar et al. 2024; Jin et al. 2023). These desirable features position Nbs as promising agents for theranostic applications, further supported by ongoing clinical trials worldwide examining their direct antitumor efficacy (Cong et al. 2024; De Pauw et al. 2023). We site-specifically functionalized our lead candidate, Nb159, using sortase-mediated ligation (Massa et al. 2016), ensuring a consistent and homogenous product with a 1:1 chelator-to-Nb ratio, which is important for maintaining binding affinity and ensuring reproducibility across batches.

The binding specificity of [^89^Zr]Zr-Nb159 to FAP was confirmed through a competitive binding inhibition in U87 tumor-bearing mice, as demonstrated by a ~ 55% reduction in tumor uptake by excess cold Nb159. The expected high renal accumulation of [^89^Zr]Zr-Nb159, a common feature of Nbs labeled by radiometals (Küppers et al. 2021), prompted us to implement two strategies to reduce renal retention. First, we engineered the construct by attaching a 20 kDa PEG moiety to increase the Nb size and its plasma circulation time. Second, co-injection with positively charged amino acid lysine to block Megalin receptors on tubular cells, further reducing renal accumulation (Roode et al. 2024). The 20 mg lysine dose was selected based on prior preclinical studies demonstrating effective renal protection without observable toxicity in mice and was evaluated here for the first time with a ⁸⁹Zr-labeled nanobody (Chatalic et al. 2015; Eerd et al. 2006).

Comparison between two radiolabeled Nb constructs, [^89^Zr]Zr-Nb159 (~ 15 kDa) versus [^89^Zr]Zr-PEG-Nb159 (~ 36 kDa), revealed distinct pharmacokinetic profiles in FAP^+^ tumor-bearing mice. As we hypothesized, the PEGylated Nb159 construct had a longer plasma residence time, with enhanced tumor retention, and much reduced renal uptake compared to Nb159, making it suitable for therapeutic applications. The co-injection with lysine had a measurable but modest effect on kidney uptake in both Nb constructs.

[^89^Zr]Zr-PEG-Nb159 further demonstrated target-specific uptake in FAP-positive U87 xenografts, which retained significantly higher signal than FAP-negative HCT116 tumors across all imaging time points. Blood pool activity was initially high, reflecting systemic distribution of the ~ 36 kDa PEGylated Nb, but declined markedly by 24 h, contributing to the strong tumor-to-background contrast observed at later imaging points at 24 and 48 h. Renal uptake remained the main clearance route, with high signal at early time points that decreased over time.

The biodistribution data confirmed minimal uptake in other organs, indicating a favorable biodistribution profile with minimal non-specific uptake. This indicates the potential of PEG-Nb159 for therapeutic applications in targeting FAP-positive tumors.

For the therapeutic study, PEG-DOTA-Nb159 was site-specifically labeled with ^177^Lu, chosen for its favorable radiochemical properties, including a long half-life (6.65 days), medium energy β-radiation, and uniform tissue distribution, making it ideal for targeted radiation delivery to the tumor while minimizing damage to surrounding healthy tissues (Pomykala et al. 2023). Administration of a single moderate dose of 18.5 MBq [^177^Lu]Lu-PEG-Nb159 resulted in statistically significant tumor growth inhibition and extended survival in mice bearing U87 xenografts, which are known to be an aggressive type of cancer cell line (glioblastoma) with poor survival. In contrast, control groups receiving [^177^Lu]Lu-DOTA or saline did not delay tumor growth, leading to earlier euthanasia of mice. Importantly, this proof-of-concept study demonstrates clear therapeutic activity even at a single dose, with no signs of toxicity or body weight loss. These findings establish a strong foundation for future optimization, including repeated dosing and combination strategies, and underscore the benefit of PEGylation in extending circulation and enhancing tumor retention, which translated directly into therapeutic benefit in FAP-positive xenografts.

While the study provides insights into the potential of the radiotheranostic agent, our study has some limitations. Future studies should perform dose escalation to establish the maximum tolerated dose of [^177^Lu]Lu-PEG-Nb159 and evaluate multi-dose regimen for clinical translation. Additionally, although PEGylation and lysine co-injection significantly reduced renal retention, histopathologic examination of kidney tissue, along with long-term monitoring of renal function biomarkers, should be incorporated to assess potential radiation-induced nephrotoxicity. This study evaluated one FAP-negative xenograft model (HCT116) as a specificity control; additional FAP-negative models could further confirm tracer selectivity across tumor types.

## Conclusion

Our study highlights the theranostic potential of our lead Nb candidate, Nb159, with a modular construct design for diagnostic and therapeutic applications in FAP-positive tumors. The radiolabeled Nb159 demonstrated specific tumor uptake for PET imaging, making it an effective companion diagnostic, while the therapeutic construct PEG-Nb159, showed significant therapeutic efficacy in U87 xenograft models. The Nb159-based theranostic pair holds promise for the detection and treatment of FAP-expressing tumors and encourages further research.

## Supplementary Information

Below is the link to the electronic supplementary material.

Supplementary Material 1.

## Data Availability

The datasets generated during and/or analyzed during the current study are available from the corresponding author upon reasonable request.
