# Supplementary material for: Developing a theranostic nanobody targeting FAP for cancer imaging and therapy
Source: EJNMMI Radiopharm Chem. 2025 Dec 29;10:82. doi: 10.1186/s41181-025-00405-z (PMC12748472; doi:10.1186/s41181-025-00405-z)
Supplement: Supplementary file 1 — Supplementary Material 1. [file 41181_2025_405_MOESM1_ESM.docx]

**Developing a Theranostic Nanobody Targeting FAP for Cancer Imaging and Therapy**

**Authors**

Lital Ben-Naim, PhD ^1,2^; Suma Prabhu, PhD ^1,2^; Miguel Ferreira, PhD ^1,2^; Shvan J. Raheem, PhD ^1,2^; Shadi A. Esfahani MD, MPH ^1,2^; Umar Mahmood MD, PhD ^1,2^; Pedram Heidari, MD ^1,2^*

**Affiliations**

^1^Division of Nuclear Medicine and Molecular Imaging, Department of Radiology, Massachusetts General Hospital, Harvard Medical School, Boston, MA 02114, United States.

^2^Center for Precision Imaging, Division of Nuclear Medicine and Molecular Imaging

Department of Radiology, Massachusetts General Hospital, Boston, MA 02129, United States.

*: Corresponding Author:

**Pedram Heidari, MD**

Service Chief, Nuclear Medicine and Molecular Imaging

Associate Director, Center for Precision Imaging

Department of Radiology, Massachusetts General Hospital

Assistant Professor of Radiology, Harvard Medical School

55 Fruit St, White 427J

Boston, MA 02114, United States

E-mail: [heidari.pedram@mgh.harvard.edu](mailto:heidari.pedram@mgh.harvard.edu)

**Supplemental Data**

**Cell Culture**

Cell cultures were maintained in a humidified incubator at 37 °C with 5% CO_2_, and each media was supplemented with 10% fetal bovine serum (FBS) and 100 U/ml penicillin, 100 mg/ml streptomycin (P/S) (Gibco). Human embryonic kidney 293T cell line (ATCC, CRL-3216) was cultured in Dulbecco’s Modified Eagle’s Medium (DMEM). Dubca (Dubai camel, ATCC, CRL-2276), human colorectal tumor CAFs (Neuromics) and human glioblastoma U87-MG (ATCC) cell-lines were cultured in Eagle's Minimum Essential Medium (EMEM) medium. The human colorectal HCT116 cell line (ATCC) was cultured in McCoy's 5A Medium (MilliporeSigma). cells were harvested using 0.05% trypsin/EDTA solution (Invitrogen).

Lentiviral Production in 293T Cells

To develop stable hFAP-expressing cells, a 3^rd^ generation lentiviral system was used as follows: 1×10^6^ 293T cells were seeded in a 15 cm culture dish and co-transfected with a mixture containing 10 µg FAP-containing vector (pLenti-CMV-Hygro harboring hFAP coding sequence, Addgene #17446), 5 µg pMDLg/pRRE, 2.5 µg pRSV-Rev, and 2.5 µg pMD2.G (Addgene #12251, #12253, and #12259) packaging plasmids. Transfection was performed using PEI reagent (Polysciences) at a 1:3 DNA:PEI ratio (μg:μg). After 48 hours, the lentivirus-containing supernatant was collected, centrifuged at 1200 rpm for 6 min, filtered through a 0.45 µm membrane, aliquoted, and stored at -80 °C.

Generation of FAP-Expressing Cells

Adherent Dubca cells were seeded at 8×10^4^ per well in 12-well plates and infected with FAP-lentiviruses. Infection was performed using a spin-fection protocol[1], where lentivirus particles were added to cells with 10 µg/ml Polybrene (MilliporeSigma) followed by centrifugation at 800×g for 90 min at 37 °C. After incubation for 6 h 37 °C, the medium was replaced with fresh EMEM. To enrich the population of high-expressing cells, fluorescence-activated cell sorting (FACS) was conducted on FAP-Dubca cells using anti-FAP PE-conjugated antibody (R&D Systems, FAB3715P) or isotype IgG1 PE-conjugated Antibody (R&D Systems, IC002P) as unstained control. Sorting was performed twice using a BD FACSAria Fusion cell sorter (BD Biosciences, MGH Pathology: Flow and Mass Cytometry Core), followed by continuous cell culture in the presence of 125 µg/ml Hygromycin to sustain stable FAP expression. Flow cytometry data were analyzed using FlowJo v10.8 software (BD Biosciences). The gating strategy is shown in Figure S1.

Construction of Nb library

Lymphocytes were isolated from peripheral blood by density gradient centrifugation using Ficoll-Paque PLUS gradient media (GE Healthcare) according to the manufacturer’s protocol. Total RNA was extracted from the lymphocytes by TRIzol (Invitrogen) and reverse-transcribed into cDNA using Verso cDNA Synthesis Kit (Thermo Fisher Scientific), from which the Nbs gene fragments were amplified in a two-step nested PCR as previously described[2]. The DNA pool of amplified Nbs was cloned into the phage-display vector pMECS and transformed into electrocompetent Escherichia coli (E. coli) TG1 cells (Lucigen) to generate a Nb library of 2.88×10^9^ individual transformants.

**Cell-based Phage-display Screening**

Phage-display screening was employed to enrich FAP-targeting Nbs from the library. TG1 clones were infected by 1×10^11^ M13K07 helper phages (New England BioLabs) to create a Nb-displaying phage library, which was then subjected to cell-based panning, adapted from Stark et al. [3] and Marsh et al. [4] with modifications. The panning rounds of phage-displayed Nbs were conducted against 8×10^5^ Dubca cells in four subsequent rounds, starting with a negative screen followed by a positive screen. For the negative screen, Nb-displaying phages were incubated with wild-type (WT) Dubca cells in a T25 flask at 30 °C for 1 h, shaking at 50 rpm. The supernatant containing unbound phages was transferred to FAP-expressing Dubca cells for a positive screen, under the same conditions. Cells were washed 3 times with 5% FBS/PBS, followed by phage elution under acidic conditions using 2 ml of Glycine-HCl/NaCl pH 2.2 for 15 min. Eluted phages were collected, centrifuged at 14,000 rpm, for 5 min, and neutralized with 50 µl of 2 M unbuffered Tris-base. Eluted phages from the fourth round were incubated with 90 µl of exponentially growing TG1 culture for 30 min, followed by plating on LB-AMP-GLU agar plates (LB agar with 100 µg/ml ampicillin and 2% glucose). Colonies were grown at 37 °C overnight and further used in a second screen.

**Selection of specific Nb binders**

To identify positive clones, 195 Nbs-containing colonies were individually evaluated for FAP binding using modified Periplasmic Extract Enzyme-Linked Immunosorbent Assay (PE-ELISA), based on [5]. Nbs extracted from periplasm were incubated with FAP-Dubca cells compared to WT Dubca cells in a 96-well plate for 1 h, at 30 °C, and screened by ELISA for binding to FAP (figure S2A). Positive Nb clones with a signal-to-control ratio higher than two were sequenced with the primers MP57 (5'-TTATGCTTCCGGCTCGTATG-3') and GIII (5'-CCACAGACAGCCCTCATAG-3') at the MGH CCIB DNA Core. Nbs sequences were grouped into 11 families based on their complementary-determining regions (CDRs) similarity.

**Cross-reactivity testing of Nbs to recombinant FAP**

The specificity of three Nbs (Nb159, Nb132, and Nb17) for human versus murine FAP was evaluated using ELISA. A 96-well plate was coated with recombinant human FAP or mouse FAP (Biolegend) and blocked with 1% bovine serum albumin (BSA) for 1 h at room temperature. Nbs were applied in triplicate as 1:10 serial dilutions (10 µM to 0.001 nM). After 1h incubation at room temperature, Binding was detected using Rabbit Anti-Camelid VHH HRP Ab (Genscript), followed by TMB substrate solution (Thermo Fisher Scientific) and termination with 1 M sulfuric acid (MilliporeSigma). Absorbance was read at 450 nm on a BioTek Cytation 5 microplate reader (Agilent).

Site-Specific Labeling with AZDye 647

Site-specific labeling of Nb-LPETGG-HHHHHH with AZDye 647 (an Alexa Fluor 647 analog, Vector Laboratories) was carried out in a reaction mixture (500 – 800 µl) consisting of 2 mg Nb 500 μM GGG-AZDye 647 (10 mM stock in DMF), 10 mM CaCl2, and 5 μM SrtA-5M (kindly provided by Dr. Mohammad Rashidian, Dana-Farber Cancer Institute) in HEPES/NaCl buffer. The reaction was incubated for 2 hours at 4 °C. Unlabeled Nb and SrtA-5M were depleted by Ni-NTA beads, and excess of unbound AZDye 647 was removed by dialysis (D-Tube Dialyzer MWCO 3.5 kDa) against PBS. The labeling was confirmed by SDS-PAGE and fluorescent gel imaging.

In vivo Optical imaging

Female athymic nude mice, 4-6 weeks old (Charles River Laboratories), were subcutaneously inoculated in the upper right flank with 2×10^6^ FAP-positive U87 or 0.5×10^6^ FAP-negative HCT116 cells in PBS/Matrigel (1:1, v/v). Once tumors reached ~200 mm^3^, mice were intravenously (i.v.) injected via tail vein with 25 µg of either Nb17, Nb132, or Nb159 (labeled with AZDye 647), or PBS (as a control), and imaged at 2, 6, and 24 h post-injection (p.i.). At the 48 h time point, mice with U87 tumor xenografts were euthanized, and organs of interest were harvested for ex vivo imaging. Fluorescent images were captured using an IVIS Spectrum in vivo imaging system (PerkinElmer) and processed using Living Image software (version 4.7.4).

**Competitive binding and biodistribution of [^89^Zr]Zr-Nb159**

To evaluate the specificity of [^89^Zr]Zr-DFO-Nb159 (~15 kDa, [^89^Zr]Zr-Nb159) as an imaging agent targeting FAP, subcutaneous FAP^+^ tumor xenografts were generated in 4-6 week-old female athymic nude mice by injection of 2×10^6^ U87 cells into the upper right flank. Once the average tumor size reached 150-200 mm^3^, the mice were randomly assigned to two groups. The blocking group was pre-treated with a 10-fold molar excess of unlabeled Nb159, and after 30 min administered with [^89^Zr]Zr-Nb159 (4.61 ± 0.54 MBq) in 100 μl of saline. The non-blocking group received a single i.v. injection of [^89^Zr]Zr-Nb159 (4.62 ± 0.233 MBq). Static PET images were acquired 1 h after radiotracer injection for 20 minutes in two-bed positions (whole-body) using the Argus PET scanner (Sedecal). Images and volumes of interest (VOIs) were processed using PMOD software version 4.4 (PMOD Technologies). At 5 h p.i, mice were euthanized, and main organs were collected (tumor, heart, spleen, liver, kidneys, lungs, intestine, brain, bone, muscle, and blood), weighted, and their radioactivity was measured in a Wizard-2 γ counter (PerkinElmer). Activity measurements were background- and decay-corrected to the time of counting, and the uptake values of the [^89^Zr]Zr-Nb159 were expressed as a percentage of injected dose per gram of tissue (%ID/g) ± standard deviation (SD).

**Comparative biodistribution of [^89^Zr]Zr-PEG-Nb159 in FAP^+^ and FAP^-^ tumors**

Female nude mice (weight: 24.8 ± 1.92 gr) bearing FAP-positive U87 or FAP-negative HCT116 tumor xenografts (200–250 mm^3^) were injected via tail vein with [^89^Zr]Zr-PEG-Nb159 (7.28 ± 0.72 MBq) mixed with 20 mg L-lysine in 100 µl total volume (n=3/group). At 24 h and at 48 h p.i. mice were euthanized, blood samples and main organs were collected, weighed, and analyzed for radioactivity on a Wizard-2 γ counter. Activity measurements were background- and decay-corrected, and %ID/g for each sample was calculated by normalizing to the total injected activity.

Supplemental Figures

**
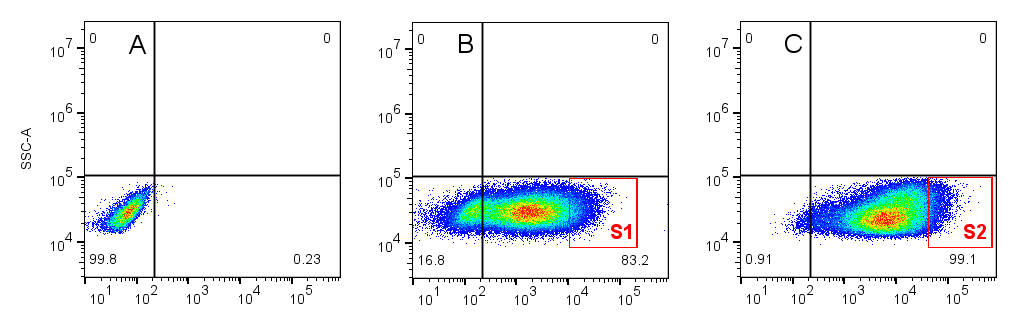

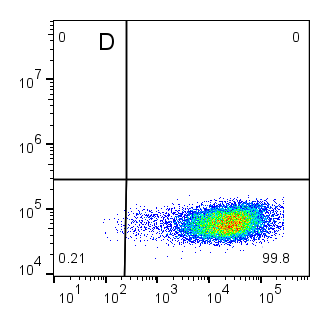
**

hFAP (PE)

**Supplemental Figure 1.** Sorting stable Dubca cells. Expression of hFAP on stable FAP-Dubca cells was assayed by FACS. A) Parental WT Dubca cells stained with PE-conjugated Isotype IgG. B-C) A population of high-expressing cells (X-axis) was collected in two rounds, i.e., S1 (8.5%) and S2 (6%) gates. D) Flow cytometry analysis of population S2.


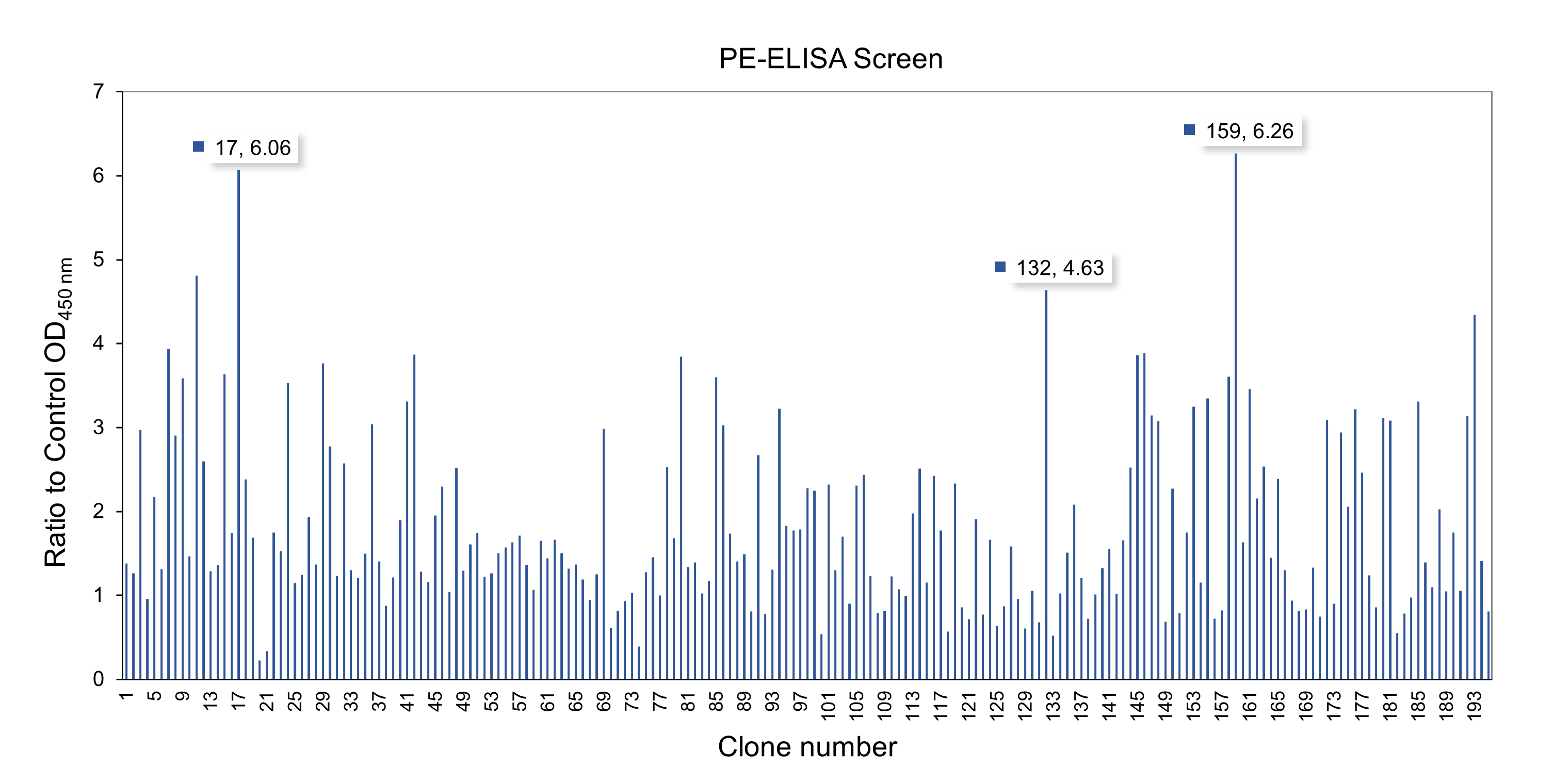


**A**

**B**

**C**

**D**

**
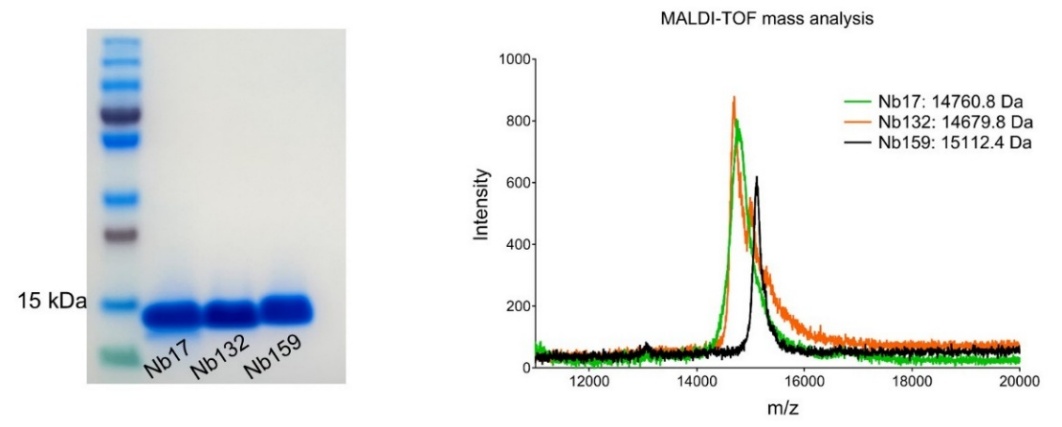
**


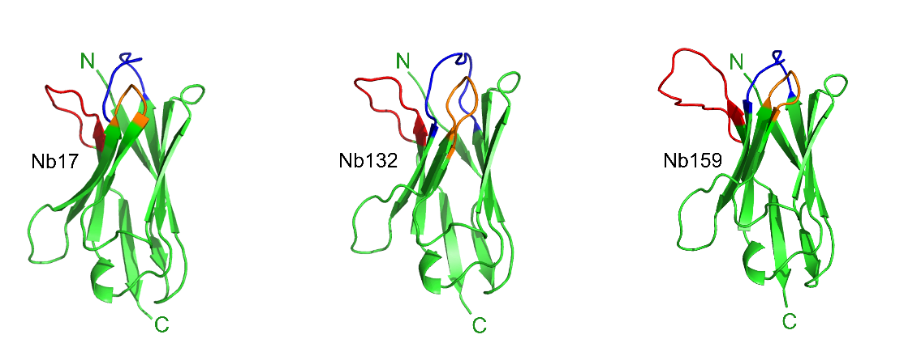


**Supplemental** **Figure 2. Nbs selection and characterization.** A) Periplasmic-extracted Nb clones binding to FAP vs. control as measured by cell-based ELISA. B) SDS-PAGE gel showing 3 highly purified proteins: Nb17, Nb132, and Nb159. C) Mass-spectrometry results confirming Nbs expected molecular mass of ~15 kDa, detected by MALDI-TOF. D) Nbs 3D structure as predicted by NanoNet [6]. The framework regions are presented in green, and the antigen binding loops CDR1, CDR2, and CDR3 are in blue, orange, and red, respectively.

**A**

**B**

**C**


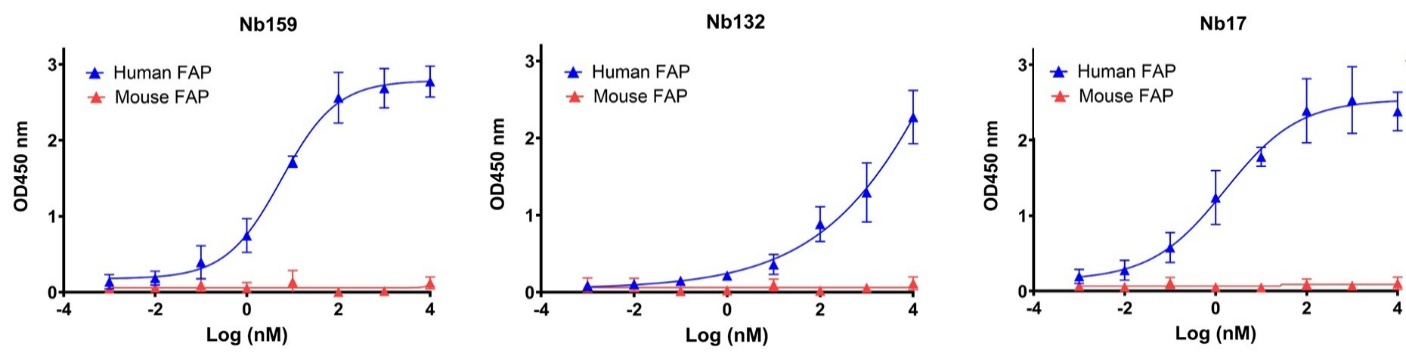


**Supplementary Figure 3. Cross-reactivity assessment of anti-FAP Nbs.** Binding of Nb159 (A), Nb132 (B), and Nb17 (C) to recombinant human and murine FAP was evaluated by ELISA (OD450 nm) across 1:10 serial dilutions (10 µM–0.001 nM). The Nbs showed specific binding to human FAP with no detectable binding to murine FAP, presented as Mean ± SD (n = 3).

**In Vivo Tumor Targeting of Labeled Nbs**

Nb-LPETGGG-AZDye 647 constructs were evaluated for tumor targeting using epifluorescence imaging (Figure S3). Nude mice bearing U87 or HCT116 xenografts were imaged at 2, 6, and 24 h following i.v. injection of labeled Nb17, Nb132, or Nb159. The images revealed rapid and selective uptake in FAP-positive U87 tumors compared to FAP-negative HCT116 tumors (Figure S3B-D). High kidney uptake was consistent with renal clearance pathways typical for molecules below the glomerular filtration threshold (~60 kDa). Ex vivo imaging of selected organs further validated the whole-body imaging results, with U87 tumors exhibiting persistent uptake of Nbs even at 48 h post-injection (Figure S4). Notably, Nb159 (K_D_ = 10.1 pM) displayed a higher tumor uptake, leading to its selection as the primary candidate for subsequent in vivo experiments.

**A**


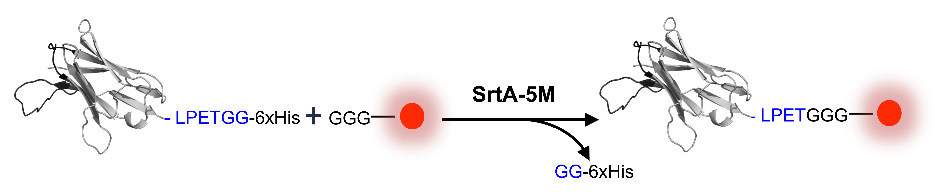


**
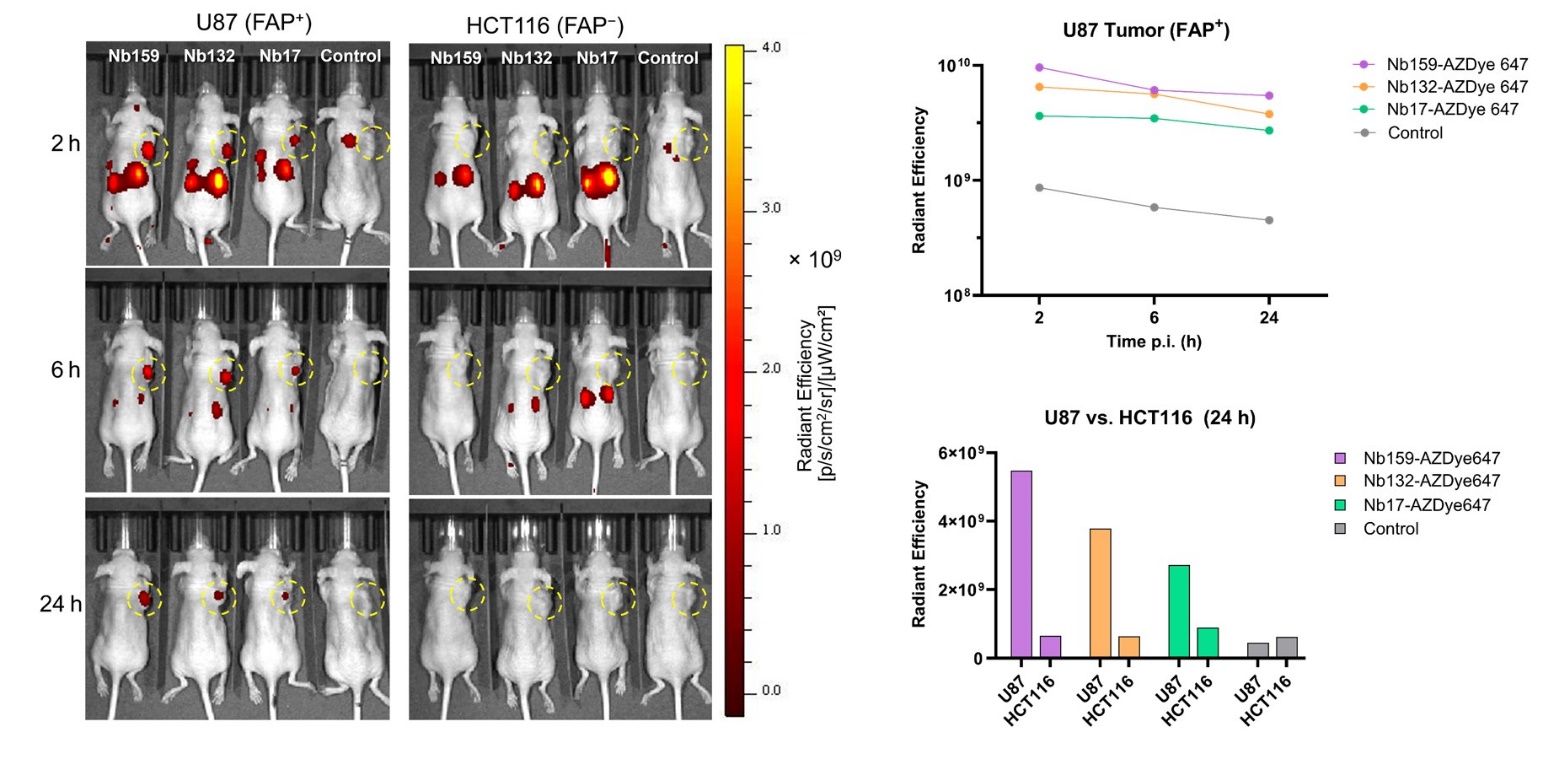
**

**D**

**B**

**C**

**Supplemental Figure 4**. **Tumor-Specific Fluorescence Imaging of Labeled Nbs.** A) Schematic representation of Nb site-specific labeling via SrtA-mediated reaction. B) In vivo epifluorescence images (λ _ex_ 640/20 nm, λ _em_ 680/40 nm) of U87 (FAP^+^) and HCT116 (FAP^−^) tumor-bearing mice at 2, 6, and 24 h post-injection of AZDye 647-labbeled Nbs (25 µg) or PBS as control (dorsal side). Anti-FAP Nb159, Nb132, and Nb17 exhibited a rapid and selective accumulation in U87 versus HCT116 tumors (right shoulder), with fast renal clearance in both groups. C) The fluorescence signal measured within U87 FAP^+^ tumor region of interest (ROI) showed a high and long-lasting specific signal starting at 2 h and slowly decreasing over time compared to the control. D) Comparison of tumor uptake at 24 h p.i. revealed higher fluorescence in U87 tumors for all three Nbs, while HCT116 tumors retained background-level signals.


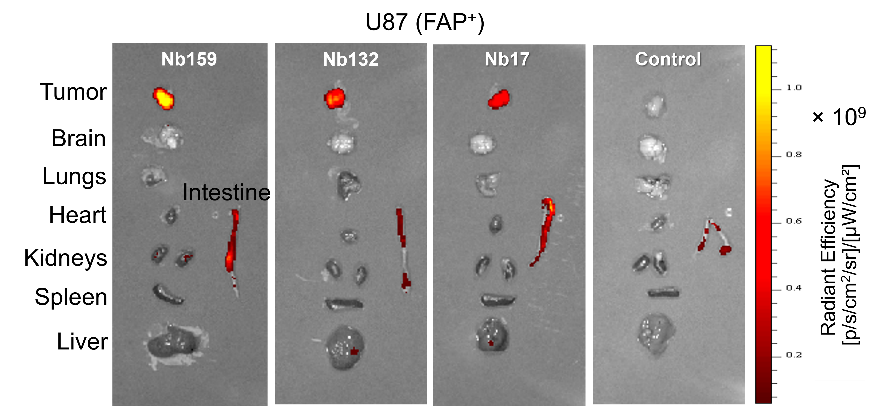


**Supplemental Figure 5. Ex vivo Fluorescent Imaging**. Tumor and main organs from U87-bearing mice were collected and imaged 48 h post Nbs administration. A fluorescence signal remained detectable in FAP^+^ tumors, while no signal was observed in non-targeted organs, except for the intestine, which displayed high background autofluorescence from ingested food across all mice.


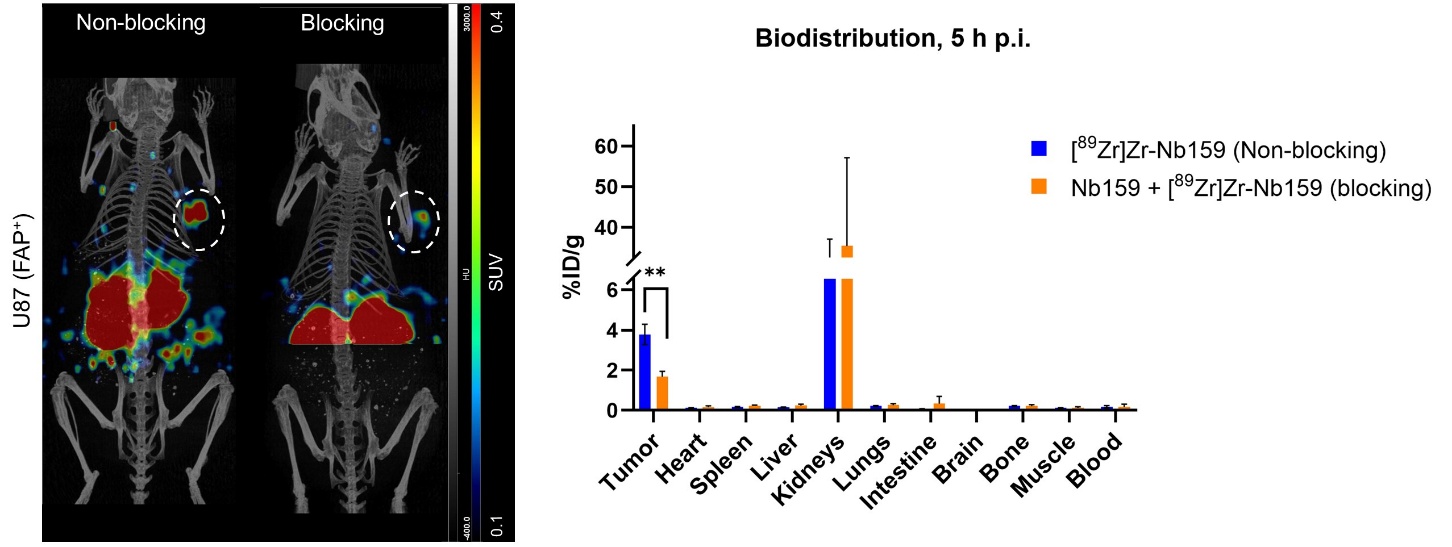


**A**

**B**

**Supplementary Figure 6. Competitive binding assay and ex vivo biodistribution**. A) Representative PET/CT images of U87 tumor-bearing mice, scanned 1 h p.i. with [^89^Zr]Zr-Nb159 (~15 kDa), with or without a blocking dose of unlabeled Nb159. The tumor uptake (marked with white dotted circle) was significantly reduced in mice receiving unlabeled Nb159. B) Ex vivo biodistribution at 5 h p.i of [^89^Zr]Zr-Nb159 in tumor and major organs of non-blocking and blocking groups. %ID/g = % injected dose per gram.

**A**

**B**

**C**


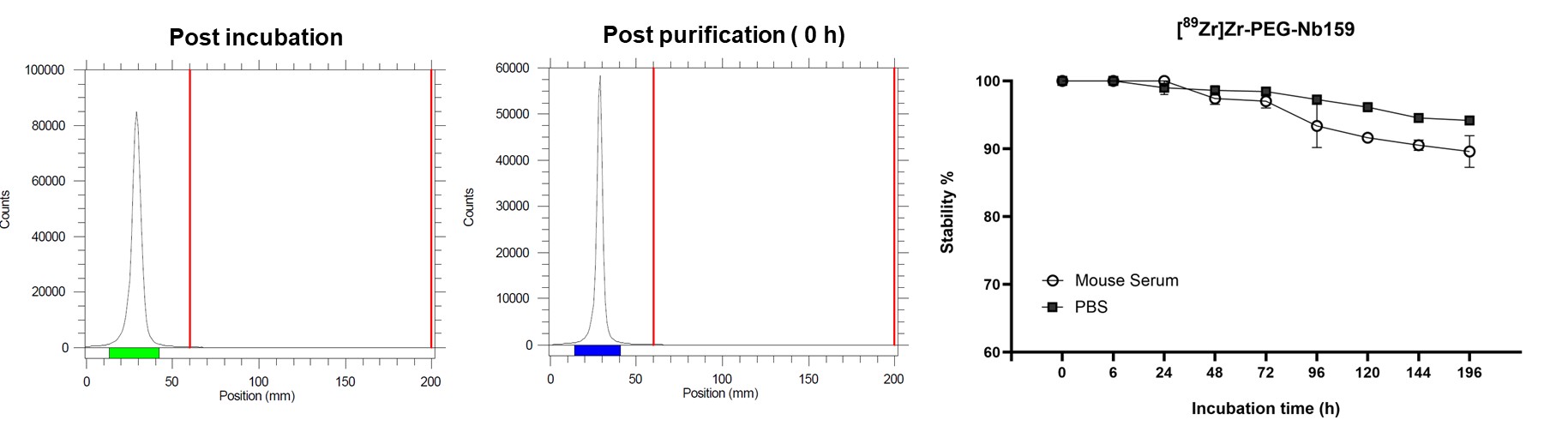


**Supplementary Figure 7. Radiochemical purity and in vitro stability of [^89^Zr]Zr-PEG-Nb159.** Representative radio-TLC chromatograms obtained (A) after incubation of ^89^Zr with PEG-DFO-Nb159 and (B) after purification by PD-10 column. Nb159 was site-specifically radiolabeled with ⁸⁹Zr, achieving a radiochemical purity of >97% and a decay-corrected yield of 90%. C) In vitro stability of [^89^Zr]Zr–PEG-Nb159 in PBS and mouse serum at 37 °C, assessed by radio-TLC over 7 days. The conjugate maintained high radiochemical integrity with >94% stability in PBS and 89% in mouse serum at day 7 (Mean ± SD).


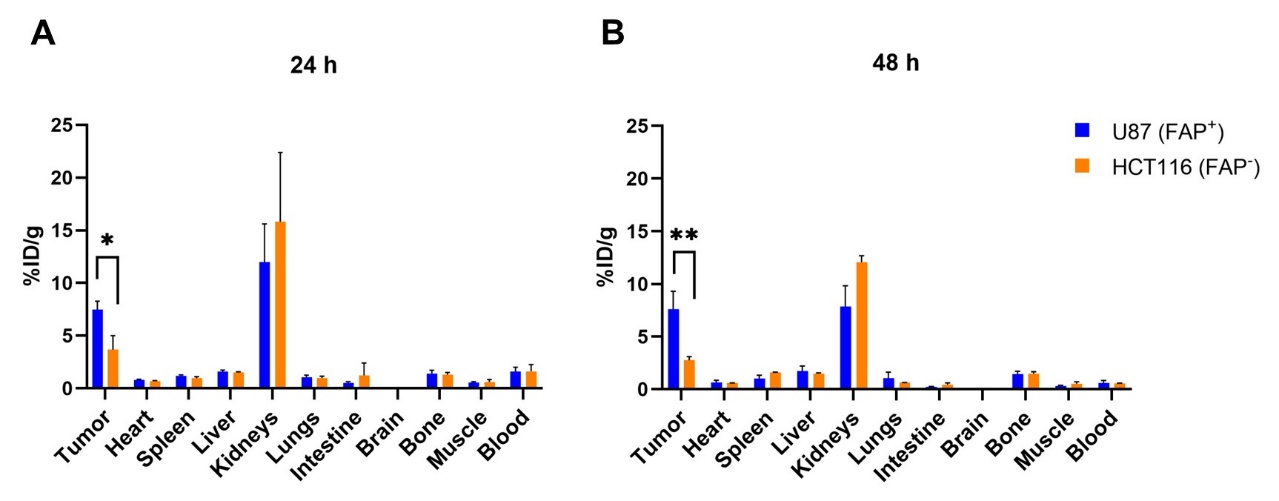


**Supplementary Figure 8.** **Ex vivo biodistribution of [^89^Zr]Zr-PEG-Nb159 in U87 and HCT116 tumor-bearing mice.** A) At 24 h post-injection, U87 tumors showed two-fold higher uptake compared to HCT116 (7.47 ± 0.80 vs. 3.67 ± 1.32 %ID/g, *P* < 0.05). B) At 48 h, the difference is more pronounced, with U87 tumors maintaining high retention while HCT116 uptake declined (7.59 ± 1.70 vs. 2.75 ± 0.36 %ID/g, *P* < 0.01). Renal clearance predominated, with kidney activity markedly decreasing from 24 h to 48 h, while activity in other organs remained moderate to low, supporting a favorable biodistribution profile and tumor specificity. Data are presented as %ID/g, mean ± SD (n=3 per group).

Supplemental References

1. Kodaka Y, Asakura Y, Asakura A. Spin Infection Enables Efficient Gene Delivery to Muscle Stem Cells. Biotechniques. 2017;63:72–6. https://doi.org/10.2144/000114576.

2. Pardon E, Laeremans T, Triest S, Rasmussen SGF, Wohlkönig A, Ruf A, et al. A general protocol for the generation of Nanobodies for structural biology. Nat Protoc. 2014;9:674–93. https://doi.org/10.1038/nprot.2014.039.

3. Stark Y, Venet S, Schmid A. Whole Cell Panning with Phage Display. Methods Mol Biol. 2017;1575:67–91. https://doi.org/10.1007/978-1-4939-6857-2_5.

4. Marsh W, Witten A, Stabenfeldt SE. Exploiting Phage Display for Development of Novel Cellular Targeting Strategies. Methods Mol Biol. 2018;1831:71–94. https://doi.org/10.1007/978-1-4939-8661-3_7.

5. Vincke C, Gutiérrez C, Wernery U, Devoogdt N, Hassanzadeh-Ghassabeh G, Muyldermans S. Generation of single domain antibody fragments derived from camelids and generation of manifold constructs. Methods Mol Biol. 2012;907:145–76. https://doi.org/10.1007/978-1-61779-974-7_8.

6. Cohen T, Halfon M, Schneidman-Duhovny D. NanoNet: Rapid and accurate end-to-end nanobody modeling by deep learning. Front Immunol. 2022;13. https://doi.org/10.3389/fimmu.2022.958584.
